# Predictors of adolescents’ mental health problems in Saudi Arabia: findings from the Jeeluna^®^ national study

**DOI:** 10.1186/s13034-017-0188-x

**Published:** 2017-09-26

**Authors:** Oraynab Abou Abbas, Fadia AlBuhairan

**Affiliations:** 1 King Abdullah International Medical Research Center and King Saud Bin Abdulaziz University for Health Sciences, Riyadh, Kingdom of Saudi Arabia; 2 Department of Pediatrics, King Abdullah Specialized Children’s Hospital, King Abdulaziz Medical City, Riyadh, Kingdom of Saudi Arabia; 3 Bloomberg School of Public Health, Johns Hopkins University, Baltimore, MD USA

**Keywords:** Adolescents, School, Mental health, Sadness, Hopelessness, Worrisome, Saudi Arabia

## Abstract

**Background:**

Depression and anxiety among adolescents require further attention as they have profound harmful implications on several aspects of adolescents’ wellbeing and can be associated with life threatening risk behaviors such as suicide.

**Objective:**

To examine the underlying risk factors for feeling so sad or hopeless and for feeling worried among adolescents in Saudi Arabia.

**Methods:**

Data from Jeeluna^®^ national survey was used. A cross-sectional, multi-stage, stratified, cluster random sampling technique was applied among a sample of students aged 10–19 years attending intermediate and secondary schools in Saudi Arabia. A self-administered questionnaire assessing several domains, including feeling so sad or hopeless and worried, was used to collect data. Logistic regression models were fitted to determine the different factors associated with mental health.

**Results:**

A sample of 12,121 students was included in this study. Feeling so sad or hopeless and feeling worried were significantly more prevalent among females and older adolescents (*p* < 0.0001). The results showed that poor relationship with parents, negative body image, and chronic illness to be significantly associated with feeling so sad or hopeless and worried.

**Conclusions:**

Symptoms suggestive of mental health problems among adolescents in Saudi Arabia are prevalent and deserve special attention. Adopting effective strategies, including regular screening and intervention programs are highly needed to better address, detect, and control early signs of these problems.

## Background

Adolescents aged 10–24 years comprise nearly 25% (1.8 billion) of the world’s 7.3 billion population. Out of the 1.8 billion, almost 9 out of 10 live in less developed countries [1]. In the Arab region, the majority of the population is below the age of 25 years [2]. Likewise, in the Kingdom of Saudi Arabia (KSA), 20% out of a 28 million population is between the ages 10–19 years [3].

Although it is believed that adolescence is a healthy time in an individual’s life, around 15% of the global burden of disease accounted for by disability adjusted life years (DALYs) is in the 10–24 years old age group [4]. During adolescence, several biological, cognitive, physiological, psychological, emotional and social changes emerge, and certain risky behaviors arise and are linked to adolescents’ health [4]. During this period, some mental health issues are also more likely to develop [5]. While mental disorders—in general—account for 45% of the burden of disease in 10–24 year olds [6], depression and anxiety are considered to be among its leading causes [7].

Mental health is an integral part of individuals’ wellbeing that is influenced not only by individual attributes or behaviors, but also by the overall social and economic circumstances and environmental factors [5]. A study by Collishaw and colleagues [8] showed a significant increase in the proportion of adolescents reporting frequent feelings of depression and anxiety where the figures had doubled between the 1980s and the 2000s.

Research on mental health problems including depression and anxiety among adolescents has found it to be associated with poor familial bonds [9], smoking [10, 11], substance use [12, 13], bullying and physical violence [14], suicide ideation and behavior [15–17], and other factors that have direct impact on adolescents’ health and wellbeing. Moreover, mental health problems during adolescence tend to persist into adulthood. Adults who suffer from depression during adolescence are at higher risk of developing major depressive disorders [18].

Although a major public health issue, adolescents’ mental health has not been granted much attention within the Arab region [19]. Gender disparities in mental health conditions are wider in the region and the burden of mental health disease is expected to continue to grow with the recent increases in regional crises over the years [19–21]. Results of the analysis of the Global School-based Student Health Survey (GSHS) that was conducted among 104,614 students from 19 low and middle income countries including Arab countries such as Jordan, United Arab Emirates, Lebanon and Morocco showed that around 35% of students reported having symptoms of depression [22]. In the KSA, individual studies have reported on the prevalence and risk factors of depression and/or anxiety [23–26] in subpopulations. The only nationally representative study addressing the prevalence of depression and anxiety among adolescents in the KSA is the Jeeluna^®^ study, in which symptoms suggestive of depression and anxiety were found to be present among 14 and 6.7% of adolescents respectively [27]. With this current study, we aim to build on previous reports and identify the underlying correlates of symptoms of depression and anxiety among adolescents in the KSA, so as to better inform policy makers, plan suitable interventions, and find sustainable solutions.

## Methods

Data for this study was taken from the 2012 Jeeluna^®^ national cross-sectional study. Jeeluna^®^ is the first national study to assess the health status and health needs of adolescents in the KSA. It was conducted during the 2011–2012 academic year using student population proportionate, stratified, multistage, cluster sampling method. The sampling occurred at the district level and the sample size per district was proportionate to the student population within that district. Male and female students aged 10–19 years, and attending intermediate and secondary schools in public and private schools throughout the 13 regions of Saudi Arabia were invited to participate in the study, yielding a school response rate of 92.5% and a student response rate of 32%. Evening schools and schools serving special needs students were excluded. Data was weighted to ensure that it was nationally representative. The detailed methodology of Jeeluna^®^ study was published earlier [27, 28].

Mental health domain is one of many other domains that were addressed in the Jeeluna^®^ questionnaire. The questions were self-reported, and most of them were guided by the GSHS as well as the Youth Risk Behavior Surveillance System (YRBSS). For the current study, different variables including socio-demographics, chronic illness, relationship with parents, and others were extracted and analyzed. Symptoms of depression, referred hereafter as “feeling so sad or hopeless” was measured with the question “*during the past 12* *months, how often did you feel excessively sad or hopeless daily for 2* *weeks or more to the extent that you stopped doing your usual activities?*”, whereas symptoms of anxiety, referred hereafter as “feeling worried”, was measured with the question “*during the past 12* *months, how often have you felt so worried about something to the extent that you stopped doing your usual activities?*”. These questions have been used in the past in adolescent health surveys [29] and are guided by the Diagnostic and Statistical Manual (DSM) of Mental Disorders criteria for identifying underlying mental health disease. Answers were categorized as most of the time/always; or never/rarely/sometimes. Those who answered always or most of the time to those questions were considered as feeling so sad or hopeless and worried.

Data were weighted to account for the probability of selection of students within each school, hence obtaining unbiased results. Statistical analysis was performed using STATA 14.

*p* values less than 0.05 were considered to be statistically significant. Descriptive statistics were obtained for the whole sample and results were reported in terms of percentages. Bivariate analysis was then performed to test for all possible associations between the dependent and independent variable. Adjusted and unadjusted odds ratios were then generated and presented.

Ethical approval for the study was obtained from the Institutional Review Board (IRB) at King Abdullah International Medical Research Center (KAIMRC), as well as the Ministry of Education. Consent and assent forms were obtained from parents and students respectively, prior to participation in the study.

## Results

### Sample characteristics

Table 1 shows participants’ demographics. For the purpose of this paper, a secondary data analysis was conducted and a total number of 12,121 observations were considered. Fifty-three percent were males, and 53% were above 15 years of age. The mean age was 15.7 ± 3.4 years. The majority of participants were Saudis (86%). Most of the students (53%) were in intermediate schools and (46%) were in secondary schools. The distribution of the students differed across the four regions in proportion to the student population per region. As for the relationship with father and mother, the majority reported having a good relationship with 84 and 93% respectively. Overall, 14% reported feeling so sad or hopeless and 6% reported feeling worried during the past 12 months prior the survey. More females (62%) and older adolescents (>15 years of age) (59%) reported feeling so sad or hopeless. The same thing applies to feeling worried that was found to be more common among females and older adolescents (63% and 66% respectively) (all *p* values <0.001). Feeling so sad or hopeless and feeling worried were more common among Saudis vs non-Saudis. On the other hand, region showed no significant association with feeling so sad or hopeless and feeling worried.

**Table 1:** Sample characteristics

Variable	N = 12,121
	% (95% CI)
Age (years)	
≤15	46.83 (41.34–52.39)
>15	53.17 (47.61–58.66)
Gender	
Male	52.97 (43.82–61.93)
Female	47.03 (38.07–56.18)
Nationality	
Saudi	86.33 (83.09–89.03)
Non-Saudi	13.67 (10.97–16.91)
Grade	
Intermediate	53.44 (46.68–60.07)
Secondary	46.56 (39.93–53.32)
Region	
Central	25.90 (22.47–29.66)
Western	32.36 (27.85–37.22)
Eastern	14 (10.33–18.70)
Northern	10.42 (6.84–15.58)
Southern	17.32 (13.70–21.66)
Chronic illness	
No	91.61 (91.0–92.18)
Yes	8.39 (7.82–9.0)
Relationship with mother	
Good	93.12 (92.37–93.79)
Neither good nor bad	5.26 (4.69–5.89)
Poor	1.63 (1.37–1.93)
Relationship with father	
Good	84.75 (83.58–85.85)
Neither good nor bad	11.2 (10.33–12.12)
Poor	4.05 (3.56–4.62)
Body image	
Happy with my body	39.65 (38.18–41.14)
Need to lose weight	45.04 (43.31–46.78)
Need to gain weight	15.31 (14.44–16.23)
Exercise during last week	
None	44.28 (41.27–47.34)
≤3 times	33.93 (32.39–35.50)
>3 times	21.79 (19.84–23.86)
Feeling so sad or hopeless	
No	85.75 (84.42–86.98)
Yes	14.25 (13.02–13.02)
Feeling worried	
No	93.34 (92.51–94.09)
Yes	6.66 (5.91–7.49)

*CI* confidence interval

### Association of feeling so sad or hopeless and feeling worried with the different variables

At the bivariate level, our results revealed that age, gender, relationship with father and mother, chronic illness, body image, and exercise to be significantly associated with feeling so sad or hopeless and feeling worried (all *p* values <0.005). Nationality was not significantly associated with symptoms of feeling so sad or hopeless, yet it was significantly associated with feeling worried (*p* < 0.05) (Table 2).

**Table 2:** Adjusted and un-adjusted odds ratios (OR) for feeling so sad or hopeless and feeling worried

	Feeling so sad or hopeless				Feeling worried			
	U-OR	95% CI	A-OR	95% CI	U-OR	95% CI	A-OR	95% CI
Age (years)								
≤15	1		1		1		1	
>15	1.35*	1.11–1.65	1.18*	1.00–1.40	1.75*	1.39–2.21	1.56*	1.25–1.94
Gender								
Male	1		1		1		1	
Female	2.08*	1.81–2.39	1.94*	1.69–2.23	2.02*	1.65–2.47	1.88*	1.56–2.28
Nationality								
Saudi	1		1		1		1	
Non-Saudi	1.07	0.90–1.29	1.11	0.93– 1.32	1.26*	1.00–1.60	1.30*	1.04–1.63
Region								
Central	1		1		1		1	
Western	0.79	0.60–1.0	0.77	0.64–0.93	0.82	0.57–1.16	0.82	0.62–1.08
Eastern	0.80	0.57–1.11	0.75	0.59–0.93	0.90	0.61–1.31	0.88	0.65–1.20
Northern	0.79	0.52–1.21	0.87	0.63–1.21	0.58	0.56–1.29	0.94	0.66–1.35
Southern	0.82	0.60–1.11	0.82	0.68–1.00	0.89	0.59–1.33	0.93	0.66–1.30
Chronic illness								
No	1		1		1		1	
Yes	2.14*	1.75–2.61	2.31*	1.86–2.87	2.74*	2.16–3.47	2.79*	2.15–3.62
Relationship with father								
Good	1		1		1		1	
Neither good nor poor	2.13*	1.77–2.56	1.77*	1.44–2.16	2.07*	1.62–2.64	1.74*	1.34–2.26
Poor	4.58*	3.65–5.72	3.44*	2.65–4.47	5.99*	4.51–7.95	4.30*	3.22–5.74
Relationship with mother								
Good	1		1		1		1	
Neither good nor poor	2.30*	1.86–2.85	1.47*	1.13–1.92	2.36*	1.76–3.15	1.47*	1.05–2.04
Poor	4.91*	3.57–6.77	2.72*	1.85–4.02	6.01*	3.95–9.17	2.64*	1.60–4.36
Body image								
Happy with my body	1		1		1		1	
Need to lose weight	1.48*	1.32–1.66	1.38*	1.22–1.56	1.45*	1.20–1.76	1.29	1.06–1.57
Need to gain weight	1.33*	1.13–1.58	1.23*	1.02–1.49	1.34*	1.07–1.69	1.13	0.89–1.44
Exercise during last week								
None	1		1		1		1	
≤3 times	0.63*	0.57–0.71	0.78	0.70–0.88	0.69*	0.58–0.83	0.88	0.72–1.08
>3 times	0.68*	0.57–0.80	0.93	0.77–1.12	0.70*	0.56–0.88	0.94	0.72–1.22

* p < 0.05

### Feeling so sad or hopeless

At the multivariate level, the associations revealed that females and older adolescents are more likely to feel so sad or hopeless with (OR 1.94; 95% CI 1.69–2.23) and (OR 1.18; 95% CI 1.00–1.40) respectively. Moreover, adolescents who had “neither good nor bad” and “poor” relationship with father had higher odds of feeling so sad or hopeless as compared to those who had a good relationship with father (OR 1.77; 95% CI 1.44–2.16) and (OR 3.44; 95% CI 2.65–4.47) respectively. The same trend continues when it comes to the relationship with mother, the poorer the relationship was, the higher the odds of feeling so sad or hopeless as compared to those who had a good relationship with mother. Adolescents who believed they needed to lose weight were more likely to feel so sad or hopeless as compared to those who were happy with their bodies. Chronic illness was also positively associated with feeling so sad or hopeless. Adolescents who self- reported having chronic illness had more than twice the odds of reporting feeling so sad or hopeless (OR 2.31; 95% CI 1.86–2.87).

### Feeling worried

In terms of statistical significance, results for the correlates of anxiety symptoms did not differ much from those of feeling so sad or hopeless. In particular, females (OR 1.88; 95% CI 1.56–2.28) and older adolescents (OR 1.56; 95% CI 1.25–1.94) were at higher odds of feeling worried as compared to males and younger adolescents. Adolescents who had a poor relationship with father or mother had respectively 4.3 and 2.64 times the risk of feeling worried as compared to those who had a good relationship with father or mother. As for nationality, our results showed that non-Saudis had higher odds of feeling worried as compared to Saudis (OR 1.3; 95% CI 1.04–1.63). Feeling of worrisome was also significantly associated with chronic illness, with adolescents with chronic illness having twice the risk of feeling worried as compared to those who were generally healthy. On the other hand, body image, exercise and region showed no statistical significance at the multivariate level.

## Discussion

The prevalence rate of depression (14%) and anxiety (6%) symptoms previously reported by the Jeeluna^®^ [27] fall within the wide range reported by different studies on depression and anxiety in the region [25, 30, 31] which made us realize the importance highlighting this issue and further investigating its underlying risk factors.

Our findings of feeling so sad or hopeless and feeling worried being more prevalent among females and older adolescents, was found to be consistent with others’ findings [32–34]. This was also supported by results reported by the analysis of the GSHS data that was conducted across 19 low and middle income countries including Morocco, Lebanon, Jordan and United Arab Emirates [22]. The higher prevalence rates among females can be attributed to different factors including genetics, biological, psychological or behavioral factors [35].

Poor family relationships or conflictual interactions within a family environment, as well as the lack of affection and support are correlated with depressive symptoms [36], as it is with other risk behaviors, such as bullying and violence [14]. This was also shown in our study, where poor relationship with mother or father was found to be significant risk factors feeling so sad or hopeless and feeling worried among adolescents in the KSA. The poorer the relationship with parents was, the higher the odds of feeling so sad or hopeless and feeling worried. Those results are aligned with the literature on this issue [26, 36, 37]. A study conducted among female adolescents in Riyadh has shown depression to be more prevalent among those who had bad relationship with their family members [26]. Other studies have also documented the importance of family roles in protecting adolescents from risky behaviors. For example, a national study about suicide ideation among adolescents in Lebanon showed that parental understanding was a protective factor against suicide ideation [15]. A regional study about adolescent and family connectedness among eight Arab societies, including Saudi Arabia, found that Arab adolescents, despite the social and cultural disparities among these societies, scored high on the connectedness to their families with females showing more connectedness than males [38]. These findings highlight the opportunity to capitalize on family relationships and connectedness and work towards focusing on building positive, strong and effective parenting and communication skills through launching awareness and educational campaigns or programs that target parents. Such programs may aim at enhancing parenting matters through equipping parents with the necessary knowledge about adolescents’ physical, emotional and mental development. After all, knowledge is a key variable in this equation; if parents are made aware of the protective impact of positive relationship with children, and their significant role in shaping their children’s well-being, they might become more engaged and more willing to take a step forward and make a difference.

Similar to other reports from different parts of the world [39–41], body image has been found to be a significant predictor of feeling so sad or hopeless and feeling worried among adolescents; those who thought they need to gain weight or lose weight were more likely to be feel so sad or hopeless. Similarly, a longitudinal study among 2139 US adolescent boys conducted between 1996 and 2009 found that distorted body image to be a risk factor for elevated depressive symptoms and tend to persist to adulthood [39]. This result is not surprising in a time where people have become deeply immersed in social media and so influenced by the ‘perfect-body’ image that may eventually affect their satisfaction with their bodies.

As for adolescents with chronic illnesses, our findings showed it to be significantly associated with mental health; similarly, a huge body of literature have documented the serious effects that chronic illness has on adolescents’ mental health [42, 43]. In their meta-analysis, Pinquart and colleagues [44] had shown that children and adolescents with chronic physical illnesses had higher levels of depressive symptoms as compared to their healthy peers. This was also documented in a study among a subpopulation of high school students in KSA, in which they found chronic illness to be a significant risk factor for depression [24].

Given the cross-sectional nature of this study, the causal inference between the dependent and independent variables cannot be established; however, our study reveals several underlying risk factors for feeling so sad or hopeless and feeling worried among adolescents in the KSA and sets the ground for more in-depth longitudinal studies that can better reflect on this situation. On the other hand, the strength of this study resides in the generalizability of the results being the first study to address mental health and associated risk factors among a nationally representative sample of adolescents in Saudi Arabia, which in turn sets a baseline for research on adolescents’ mental health in the country. Adolescents’ mental health is an important issue that is, unfortunately, being widely underestimated. A huge body of research unveils the deleterious effects that depression during adolescence has on their wellbeing, not only as adolescents but as future adults too. This can be avoided through early detection of symptoms when present and effective school and community-based interventions that are tailored to the Saudi cultural context where, as in many other Middles Eastern countries, mental health problems are still stigmatized [45]. Accordingly, mental health interventions in the Middle East should take into account the fundamental role of families, adolescent-family connectedness and stigma associated with mental health [38, 46].

Ministry of Health and School Health Programs should work hand in hand in planning public awareness campaigns and training programs that target adolescents, parents and teachers. Parents should be well educated about the importance of positive communications with adolescents and this should not be difficult for an Arab country like Saudi Arabia, where family ties are highly cherished and families are considered to be the first line of support and protection.

Annual screening for depression, as recommended by the United States Preventive Services Task Force [47] should be implemented in schools. Effective professional counseling services should be implemented at all schools to help support students better cope with their problems, be it social, emotional or behavioral. Attention to capacity building in adolescent health, including adolescent mental health, is much needed [48] and will provide healthcare providers with the necessary knowledge, awareness, and skills for addressing adolescents’ health needs. Though some attention to this has begun with the first Adolescent Health and Medicine Capacity Building Workshop in the Region in 2016 (AlBuhairan, unpublished), much more is needed. Lastly, the civil society should also be held accountable for planning prevention programs that promote positive mental health and creating a supporting environment so as to overcome shame and stigma linked to mental health.

## Conclusions

Mental health issues are a major public health concern that have serious implications on adolescents’ wellbeing. This study reveals the underlying risk factors of symptoms of depression and anxiety among adolescents in Saudi Arabia and highlights the importance of taking the necessary actions and planning suitable interventions that can lessen its harmful impact if not preventing it. Further in-depth research studies that assess adolescents’ mental health using diagnostic tools for depression and anxiety are needed. Also, parents–adolescents research in Saudi Arabia is missing and requires closer investigation.

## Abbreviations

CI: confidence interval
KSA: Kingdom of Saudi Arabia
OR: odds ratio
PV: physical violence
GSHS: Global School-based Student Health Survey
YRBSS: Youth Risk Behavior Surveillance System
